# Evaluation of total oxidative stress and antioxidant capacity of brain tumour patients attending referral hospitals in Addis Ababa, 2020: a comparative cross-sectional study

**DOI:** 10.3332/ecancer.2022.1391

**Published:** 2022-05-16

**Authors:** Fitalew Tadele Admasu, Biruk Demissie, Getachew Yideg Yitbarek, Zeleke Geto, Aragaw Tesfaw, Edget Abebe Zewde, Animut Tilahun, Gashaw Walle, Tigist Tefera Bekele, Mezgebu Legesse Habte, Teka Obsa Feyisa, Tadeg Jemere Amare, Wubet Alebachew, Sintayehu Asnakew, Ermiyas Sisay, Markeshaw Tiruneh, Getaneh Atikilt Yemata, Tigabu Munye Aytenew, Tadesse Asmamaw Dejenie

**Affiliations:** 1 Department of Biomedical Science, School of Medicine, College of Health Sciences, Debre Tabor University, Debre Tabor, 272, Ethiopia; 2 Department of Public Health, College of Health Sciences, Debre Tabor University, Debre Tabor, 272, Ethiopia; 3 Department of Biomedical Sciences, School of Medicine, College of Health Sciences, Wello University, Wello, 1242, Ethiopia; 4 Department of Biochemistry, College of Biomedical Sciences, School of Medicine, College of Health Sciences and Medicine, Haramaya University, Harer, 138, Ethiopia; 5 Department of Nursing, College of Health Sciences, Debre Tabor University, Debre Tabor, 272, Ethiopia; 6 Department of Psychiatry, School of Medicine, College of Health Sciences, Debre Tabor University, Debre Tabor, 272, Ethiopia; 7 Department of Biochemistry, School of Medicine, College of Health Sciences and medicine, Gondar University, Gondar, 196, Ethiopia

**Keywords:** brain tumour, oxidative stress, tumour metabolism, tumour grade, Ethiopia

## Abstract

**Background:**

The exact cause of brain tumours is still unknown, but disruptions of redox balance are thought to play a significant role in all stages of brain tumour development. However, the roles of free radical imbalance at different grades of brain tumour and degree of oxidative stress before and after surgery have not been addressed in prior studies.

**Aim:**

A comparative cross-sectional study was conducted to assess the redox imbalance among confirmed brain tumour patients.

**Methods and results:**

An institution-based comparative cross-sectional study was conducted on a total of 100 participants (50 brain tumour patients and 50 controls) at referral hospitals in Addis Ababa, Ethiopia. Descriptive statistics, *t*-test and analysis of variance (ANOVA) (*post-hoc*) analysis were used and statistical significance was declared at *p* ≤ 0.05. The serum oxidised glutathione and total oxidative stress were significantly higher in the serum of brain tumour patients (0.72 ± 0.03 μM/μg and 9.66 ± 1.76 μmol H_2_O_2_ Eq/L, respectively) compared to the control group (0.21 ± 0.07 μM/μg and 6.59 ± 0.81 μmol H_2_O_2_ Eq/L, respectively) (*p* ≤ 0.05). The serum total oxidant status gradually increased as the tumour grade increased, being higher in grade four (11.96 ± 0.72) and lower in grade one (8.43 ± 1.56), and the mean differences were statistically significant (*p* ≤ 0 05). A statistically significantly higher total antioxidant capacity (116.78 ± 5.03 Trolox Eq/L) was obtained in the post-surgery than pre-surgery level (79.65 ± 17.914 Trolox Eq/L) (*p* ≤ 0 05).

**Conclusion:**

Higher oxidant and lower antioxidant levels were found in the serum of brain tumour patients than in the control group. The post-surgery oxidant level was lower than the pre-surgery state. The findings of this study could suggest that redox imbalance may have a role in the pathophysiology of brain tumours, but further experimental studies are needed.

## Introduction

Cancer cells show complete alterations of cellular physiology, metabolism, genome and proteome to continuously divide and avoid control of the cell cycle [1]. Disruption of redox balance and an increase in production or a defect in the removal of free radicals play a significant role in tumour initiation and progression by affecting tumour cell growth and survival through molecular damage, changes in cellular pathways and signalling and modulation of the cell cycle [2–4].

Generally, brain tumour is rare disease and its overall lifetime chance of developing is less than 1%. Although brain tumours are rare, they cause morbidity and mortality disproportionate to their incidence [1, 5, 6]. The world age-standardised incidence rate of primary brain tumours ranges from 4.3 to 18.6 per 100,000 per year. Primary brain tumour accounts for 1.4% of new cancer diagnoses in the USA and 2.7% of deaths due to cancer [7]. Northern Europe and the USA are countries with the highest prevalence and India and Southeast Asia are countries with the lowest prevalence of brain tumours worldwide [8]. More than any other cancer, brain tumours have long-lasting and life-altering physical, cognitive and psychological impacts on a patient’s life, and survival is generally poor compared to many other cancers [9].

Brain tumours are a mixed group of mass or growth of abnormal cells originating from intracranial tissues and meninges affecting different brain cell types and are an important cause of morbidity and mortality in both adults and children [10, 11]. The cause of brain tumours is still largely unknown, but some genetic and environmental factors may contribute to the development of brain tumours and the risk factors are much less defined than for other types of human cancers [6] Astrocytic tumour group, oligodendroglial, mixed gliomas and malignant meningiomas are the most common histological types of brain tumours worldwide [8].

Because of the brain cells’ high energy and oxygen consumption, abundant free radical-sensitive polyunsaturated fatty acids [12], high membrane surface area to cytoplasmic volume ratio, excitotoxic glutamate, high Ca^+2^, autooxidation of neurotransmitters, continuous formation of iron, low level of antioxidant defence particularly catalase, glutathione peroxidase, vitamin E, cytochrome P450 and O_2_ free radicals from neuronal mitochondria, the brain tissue is highly vulnerable to oxidative stress-mediated injury compared to other parts of the human body [13, 14]. Oxidative stress, a disturbance in the oxidant–antioxidant balance, is known in conjunction with the initiation and progression of brain tumour and the acquisition of almost all tumour cell hallmarks, including sustained cell proliferation, immortalisation, cell death escape, metastasis, recurrence and chemoresistance [15]. Generally, lower levels of antioxidant molecules are found in brain tissue, and in brain tumour cells, the expression of major antioxidant enzymes is altered and the anti-proliferative tumour suppressor activity of antioxidants may be lost [16].

In Ethiopia, there is far less cancer research due to resource limitations, especially brain tumours have been largely underestimated or ignored and not even listed among the important cancers. In this perspective, this study aimed to assess the redox imbalance, reduced and oxidised glutathione and oxidative stress index (total oxidant status/total antioxidant capacity (TOS/TAC)) of brain tumour patients attending public referral hospitals in Addis Ababa, Ethiopia.

## Materials and methods

### Study design, setting and period

An institution-based cross-sectional study was implemented on a total of 100 participants. The study participants were recruited from the only four hospitals providing neurology and/or neurosurgery service in Ethiopia and one (lideta) health centre for selection of the control group in Addis Ababa, Ethiopia. The study was conducted from January to November 2019; accordingly, 50 brain tumour patients and 50 apparently healthy individuals as a control group were recruited.

### Study participants

**Brain tumour cases**: All pathologically confirmed adults (above 18 years) who were brain tumour patients of both sexes and visited the pathology, oncology and neurosurgery departments of the hospitals during the data collection period were eligible for the study. Spinal cord tumours, metastatic brain tumours, primary brain lymphomas, patients with additional conditions, such as other malignancies, advanced organ failure or active infection, were excluded.

**Control group**: To analyse and compare the oxidant and antioxidant statuses of brain tumour patients, apparently healthy volunteers were recruited as a control group. The control group comprised all apparently healthy clinical and administrative staff members who were working at Lideta health centre during the data collection period. After screening (detailed medical history and physical examination) for any acute febrile illness and chronic disease, including known cancer, brain CT scan imaging was performed for any brain lesion. Finally, individuals who fulfilled the selection criterion and with no brain lesion were included (sex and age range matched). Accordingly, 50 brain tumour patients and 50 individuals as a control group participated.

### Data collection procedures

### Blood sample collection procedure and laboratory analysis

Blood samples for laboratory analysis were taken once from the control group (*n* = 50) and twice from the brain tumour patients: on admission before surgery (*n* = 50) and 30 days after surgery (*n* = 43). Before sample collection, informed consent was obtained after explaining the objective and aim of the study, then before and after surgery for cases and once after 1 day of fasting for control group around 3–5 ml of venous blood samples were collected. Blood samples and participants’ responses to questionnaires were collected by experienced professional laboratory technologists and nurses. Accordingly, a total of 143 blood samples (93 cases and 50 from the control group) were collected using a serum separator tube. Serum samples were harvested in Eppendorf tubes after centrifuging the blood at 4,000 rpm for 5 minutes and stored at a −80°C in a deep freezer until laboratory analysis. The different serum parameters were determined using the kits and chemicals bought from Sigma-Aldrich, Merck and BDH Chemical Company, USA, and laboratory analysis was carried out at Tikur Ambessa’s biochemistry lab [17].

### Total glutathione (GSH) assay principle

The total reduced glutathione in the serum sample was determined using the principle of Rahman *et al* [18]. The assay starts with the oxidation of GSH by the sulfhydryl reagent 5,5′-dithio-bis(2-nitrobenzoic acid) to form a yellowish derivative of 5′-thio-2-nitrobenzoic acid (TNB). The formed TNB was measured at 412 nm and proportional to the concentration of GSH in the sample [18].

### Oxidised glutathione (GSSG) assay principle

The oxidised glutathione of our sample was also determined based on the principle of Rahman *et al* [18]. The principle used NADPH and GSSG reductase recycling method, and by measuring NADPH spectrophotometrically at a wavelength of 340 nm, the amount of GSSG was determined [18].

### TOS assay principle

The total oxidants present in the serum sample were determined by using the principle of Erel [19]. The process which uses dilute acid, H_2_O_2_, tert-butyl hydroperoxide, cumene hydroperoxide and protein hydroperoxide oxidises ferrous ions present in the solution to ferric ions. The reaction is upgraded by glycerol molecules, and then a coloured compound is formed when the ferric ion reacts with xylenol orange in an acidic medium. The absorbance which is measured spectrophotometrically at 560 nm wavelength is correlated to the total oxidant molecules present in the plasma. The outcomes were expressed as μmol H_2_O_2_ equivalent/L [19].

### TAC assay principle

The total antioxidant capacity of our serum sample was obtained based on the principle of Koracevic *et al* [20]. The principle works by using the capacity of the serum sample to inhibit the production of thiobarbituric acid reactive substances from sodium benzoate under the presence of the free oxygen radicals derived from Fenton’s reaction. A yellowish brown colour is formed upon the addition of samples; the oxidants present in the reaction are scavenged by the antioxidants present in the sample, giving a viable estimation of TAC and the result was expressed as mmol Trolox equivalent/L [20].

### Determination of oxidative stress index (OSI)

The ratio of total oxidative stress to total antioxidant capacity, called OSI, was calculated based on the method described by Erel [19]. After converting the resulting unit of TAS to µmol/L, the OSI was calculated using the following formula:


$$\mathrm{OSI}\;(\mathrm{arbitrary}\;\mathrm{unit})=\frac{\mathrm{TOS}(\mu \mathrm{mol}\;H_{2}O_{2}\;\mathrm{equivalent}/L)}{\mathrm{TAC}(\mu \mathrm{mol}\;\mathrm{Trolox}\;\mathrm{equivalent}/L)}\times 100$$

### Tumour grading and histopathological determination

During the pathological examination of the brain tumour tissue, histologic characterisation of the tumour and tumour grading were performed. The 2016 WHO central nervous system (CNS) tumour grading system was used; accordingly, tumours were graded from grade I to IV. Tumour size was measured from brain tumour patient’s radiographic (CT scan or MRI) images.

Assigning brain tumours to a specific grade, especially to those tumour types having no 2016 CNS tumour grading, was the major challenge during the study. Therefore, we made some rearrangement based on the histologic feature of the tumour where schwannomas were graded as grade one, medulloblastomas were graded as grade four and ependimomas were graded as grade three.

### Data analysis procedures

Before analysis, the data were checked for completeness and internal consistency, then it was coded and entered using Epi Info version 7.2 and analysed using SPSS version 23. Descriptive statistical analysis was used to present the socio-demographic and clinical characteristics of the study participants. An independent sample *t*-test was used to compare the different serum parameters between cases and the control group and one-way ANOVA (*post-hoc* analysis) was used to compare serum parameters between different brain tumour grades. Pearson’s correlation was used to describe the final model. *p*-values less than 0.05 were used to identify statistically significant results.

### Data quality assurance

Before actual data collection, training and discussion with data collectors, neurosurgeons and pathologists were carried out. The blood samples were taken using aseptic techniques with the standard operational procedure. The kit was made free from contamination and checked for consistency. All laboratory procedures were handled by professional laboratory technologists and the results were checked for completeness on a daily basis by an immediate supervisor.

## Results

### Socio-demographic characteristics of study participants

In this study, a total of 100 participants (50 brain tumour patients and 50 healthy volunteers as the control group) were included. The mean age of brain tumour cases and healthy controls was 35.25 and 36.2 years, respectively. None of the brain tumour cases (0/50) and 7/50 (14%) controls smoke, and 26/50 (52%) brain tumour patients and 14/50 (28%) controls drink alcohol. Most of the brain tumour patients (31, 62%) and 27 (54%) controls had no exercise habit at all (Table 1). There were no statistically significant differences between cases and the control group in terms of BMI grouping and physical exercise habits (*p* > 0.05).

### Histological and pathological characterisation of brain tumour tissue of brain tumour patients

In this study, 50 histologically confirmed brain tumour patients were included. The 2016 WHO CNS tumour classification and grading, which uses histological features of the tumour under light microscopy, the genetic basis of tumourigenesis and the molecular marker, was used for tumour grading [21]; subsequently, tumour tissue was graded into grade I, grade II, grade III and grade IV (Table 2). Twenty-one tumours were of grade one and six were of grade four. The mean tumour size (longest diameter) as it appears on CT or MRI was 3.6 ± 1.12 cm (Table 2).

### Biochemical analysis

### Serum level of glutathione (GSH and GSSG)

The mean serum level of GSH of brain tumour patients was significantly lower compared to the control group (11.09 ± 1.26 versus 20.04 ± 2.6 μM/μg) (*p* < 0 05) and the mean serum GSSG of brain tumour patients was significantly higher than that of the control group (0.72 ± 0.03 and 0.21 ± 0.07 μM/μg, respectively) (*p* ≤ 0.05) (Table 3).

### Serum levels of TOS and TAC

The serum TOS level of brain tumour patients (9.66 ± 1.76 μmol H_2_O_2_ Eq/L) was significantly higher than that of the control group (6.59 ± 0.81 μmol H_2_O_2_ Eq/L) (*p* ≤ 0.05). In the serum samples of brain tumour patients, there was a significantly lower concentration of TAC (79.65 ± 30.3 mmol Trolox Eq/L) than that of the control group (118.68 ± 10.19 mmol Trolox Eq/L) (*p* ≤ 0.05) (Table 3).

### Oxidative stress index (OSI)

Likewise, serum samples of brain tumour patients had significantly higher OSI values (13.37 ± 5.89 arbitrary unit) than the control group (5.61 ± 0.92 arbitrary unit), and the difference was statistically significant (*p* = 0.006) (Table 3).

### Comparison of the different serum parameters (analysis of variance) among the different tumour tissues grades

**Figure 1.**
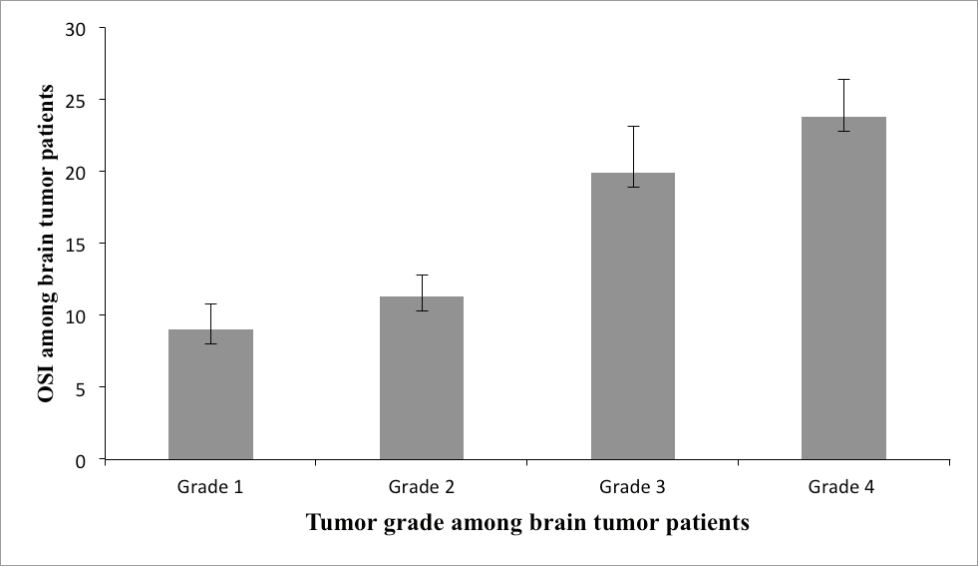
The serum oxidative stress index across different tumour grades identified among brain tumour patients.

The different serum parameters were measured and compared across the four tumour tissue grades of brain tumour patients (Table 4) (Figure 1). The mean serum GSH level was higher in grade one (11.96 ± 1.13 μM/μg) and lower in grade 4 (9.46 ± 0.46 μM/μg) and statistically significant mean differences were obtained between grades four and three and between grades one and two (*p* ≤ 0 05). Serum GSSG was higher in grade three (0.75 ± 0.029 μM/μg) and lower in grade one (0.69 ± 0.02 μM/μg) and statistically significant mean differences were observed between grades one and two and between grades three and four (*p* ≤ 0 05).

The serum TOS concentration gradually increased as the tumour grade increased, being higher in grade four (11.96 ± 0.72 μmol H_2_O_2_ Eq/L) and lower in grade one (8.43 ± 1.56 μmol H_2_O_2_ Eq/L), and statistically significant mean differences were observed between grades one and two and between grades three and four (*p* ≤ 0 05). A statistically significant mean difference was observed across all tumour grades in the serum TAC and it was higher in grade one (93.80 ± 4.49 mmol Trolox Eq/L) and lower in grade four (50.55 ± 3.14 mmol Trolox Eq/L) (*p* ≤ 0 05) (Table 4) (Figure 1). Correlation analysis also revealed that OSI showed a positive and significant correlation with tumour grade of brain tumour patients (*r* = 0.90, *p* = 0.001).

### Comparison of the different serum parameters at different time periods among brain tumour patients

**Figure 2.**
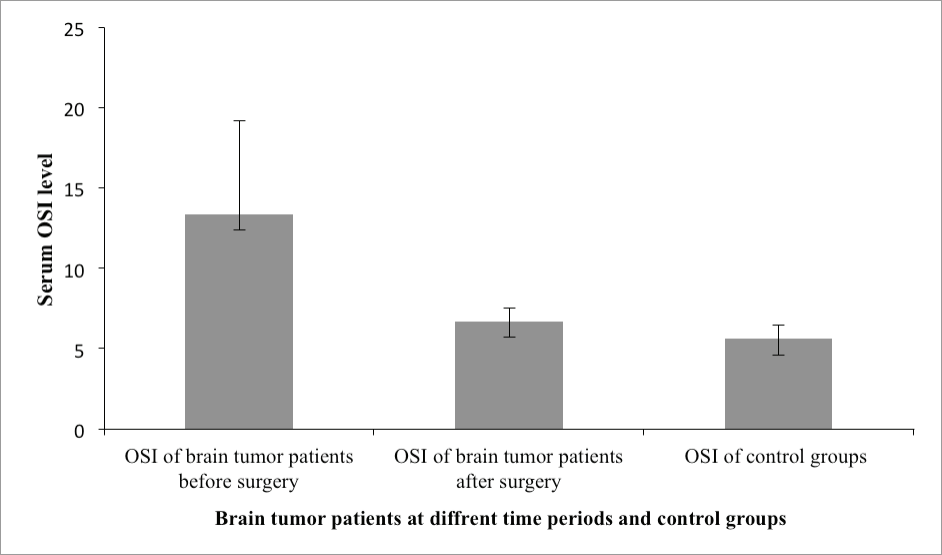
The serum oxidative stress index among brain tumour patients at different time periods and control group

Finally, our study also tried to assess the concentration of serum oxidants and antioxidants of brain tumour patients who survived 1 month after intracranial surgery (*N*= 43) and was compared with serum values taken before surgery and with control group (Table 5) (Figure 2).

The mean post-surgery concentration of GSH (17.36 ± 2.55 μM/μg) was higher than the pre-surgery time (11.09 ± 1.26 μM/μg) but lower than that of the control group (20.04 ± 2.6 μM/μg), and a statistically significant mean difference was obtained across the control group, before surgery and after surgery (*p* ≤ 0 05). Serum GSSG was higher in brain tumour patients before surgery (0.72 ± 0.032 μM/μg) but decreased after surgery (0.47 ± 0.08 μM/μg), and the mean difference across all the three comparing groups was statistically significant (*p* ≤ 0 05).

The mean post-surgery serum TOS concentration (7.79 ± 0.92 μmol H_2_O_2_ Eq/L), although it was higher when compared to the control group, it was lower from the pre-surgical level (9.66 ± 1.76 μmol H_2_O_2_ Eq/L), and the mean difference was also statistically significantly across the comparison groups (*p* ≤ 0 05). Similarly, the post-surgery TAC concentration of brain tumour patients (116.78 ± 5.034 mmol Trolox Eq/L) was increased from the pre-surgery level (116.78 ± 5.03 mmol Trolox Eq/L) but was lower than the control group (118.68 ± 10.19 mmol Trolox Eq/L), and the mean difference was statistically different only between the pre- and post-surgery groups (*p* ≤ 0 05). Finally, a statistically different mean of OSI was observed between the pre- and post-surgery groups (*p* ≤ 0 05) (Table 5).

## Discussion

Current therapeutic strategies of brain tumours include surgical resection, radiotherapy and/or chemotherapy. However, because of intrinsic and acquired molecular resistances, many brain tumour patients fail to benefit from these therapeutic strategies. Therefore, searching for an alternative molecular treatment method and improving existing strategies are urgent and necessary [22]**.** The role of redox imbalance in brain tumour development through their association with apoptosis, oncogene expression, activation of aberrant cell signalling cascades, enhanced metabolism and mitochondrial dysfunction is significant [23, 24].

In our study, serum levels of GSH, GSSG, TOS, TAC and OSI were determined in search of potential treatment and prognosis target for brain tumour disease.

The current study shows that the serum GSSG of brain tumour patients was significantly increased and the counterbalancing GSH significantly decreased when compared to the control group. These findings agree with the reports of Liu *et al* [25], Gamcsik *et al* [26], Tadele *et al* [27] and Zhu *et al* [22]. Liu *et al* [23] reported that GSSG levels in primary brain tumours were more than twice the level found in healthy individuals. In another group, Gamcsik *et al* [26] reported that oxidised glutathione levels among brain tumour patients show a significant increment compared to healthy individuals. The possible justification could be due to unusual high free radical production in brain tumours as reduced glutathione in healthy brain tissues normally is below 5% [22, 28]. Mutations in glutathione (GST) enzymes, depletion of GST by overproduction of toxic substances and high activity of the glutathione-degrading enzyme could substantially reduce the protective activity and may increase the susceptibility of the brain to tumour development, suggesting that GST and GST genes are important molecules associated with the development and treatment of brain tumours [25, 26].

When cells have higher production of reactive oxygen species than cellular antioxidant defences, toxic substances develop inducing oxidative stress [29–31]. The current study’s results show that the serum TOS of brain tumour patients was significantly elevated and the counterbalancing TAC was significantly decreased when compared to the control group. These findings agree with the findings of other studies [12, 27, 30, 32]. The possible justification could be due to elevated metabolic activity in tumour cells, tumour cells oxidant production, especially H_2_O_2_, oncogene activation, increased cellular receptor signalling, mitochondrial dysfunction [33] and further free radical production by exogenous free radical-generating carcinogenic insults, such as cigarette smoke, heavy metals, ionising radiation and asbestos [24, 30]. In addition, in brain tumour patients, oxidative stress can also arise from the unusual generation of free radicals and low expression or inactivation of antioxidant defence by mutations of tumour suppressor genes, like mutant BRCA1 and p53, and attenuated activation and function of nuclear factors [34–36]. These all lead to oxidative insults to DNA and reduced DNA repair [37], which may promote tumour cell proliferation, metastasis and progression [33, 38–40]. Brain tumour patients also had a significantly higher ratio of TOS to TAC (OSI) than the control group. OSI is a more comprehensive non-invasive method to evaluate oxidants, and enzymatic and non-enzymatic antioxidant molecules provide indirect information about the redox status [41].

Tumour grade is considered a highly valuable prognostic factor in brain tumour patients, followed by the location and type of brain cells involved; the lower the tumour grade the better the prognosis, and higher tumour grades are often associated with significantly poor clinical outcomes [42, 43]. Our study has found a different redox status even within the different tumour grades of brain tumour patients. The mean GSH gradually decreases as the tumour grade increases and the mean serum GSSG level fluctuates across the tumour grades, but the concentration was higher in the higher tumour grades than the lower ones. The mean TOS gradually increased as the tumour grade increased and the counterbalancing TAC gradually decreased as the tumour grade increased. Oxidative stress appears to promote and regulate brain tumour growth by promoting tumour angiogenesis and tumour cell proliferation, limiting tumour cell apoptosis, and it may also improve tumour survival and self-renewal by targeting protein kinase A and Notch signalling pathways [44–46]. Similar findings were also reported in other previous studies on different cancer types like in patients with follicular lymphoma and lung cancer suggesting that besides tumour initiation, oxidative stress might also have a role in tumour promotion and progression [47].

Finally, our study evaluated the post-interventional serum parameters in those brain tumour patients who survived 1 month after intracranial surgery (*N* = 43) and tried to see changes from the pre-surgical state and were also compared with serum values of control group.

The mean post-surgery GSH was higher than the pre-surgery group but lower than that of the control group, and the serum GSSG decreased after surgery. Likewise, the mean post-surgery serum TOS concentration, although it was higher when compared to control group, it was lower from the pre-surgical level and the post-surgery TAC concentration increased from the pre-surgery level but was lower than the control group. To our knowledge, there are no other research studies carried out to elucidate the pre- and post-intervention redox status of brain tumour patients, but significantly higher GSH levels were found after surgery than before the surgery in liver cancer patients [48]. Glutathione (GSH) is considered to be one of the leading detox agents and the level of GSH can influence the sensitivity of cells to anticancer treatment and toxicity [22].

The current treatment strategies of brain tumours are based on surgical excision with/without radiotherapy and chemotherapy, but tumour recurrence with the acquisition of resistance and aggressiveness by activation of several systems, including modification of redox equilibrium, is a major concern [49]. The imbalance redox state can result in therapy resistance, modulate the efficacy of treatments, drug sensitivity, induction of inflammation, angiogenesis and mainly enzymes that stimulate the production of ROS and their targets will be up-regulated [27, 41, 50–54]. Therefore, the use of different antioxidants that modulate the metabolism and ROS production by specific synthetic and natural dietary constituents, such as phytoestrogens, flavonoids, polyunsaturated fatty acids and vitamins, may have anticancer actions against brain tumour [49]. Furthermore, antioxidant supplementation may decrease the tumour aggressiveness and gliomagenesis progression [55].

## Limitations of the study

Despite being the first of its type and the robust methodology we employed, the study was not without limitation. The main limitation of this study was the tumour grading system; although we have used the currently applicable brain tumour grading system (The 2016 CNS tumour grading system), some tumour types included in our study (like schwannomas, medulloblastomas and ependimomas) were not in the system and therefore were graded based on their histologic features. The other limitation was being a cross-sectional study, and due to the high cost of reagents and supplies, the study was conducted with a small sample size and especially the study’s conclusion may be influenced by a small sample size of patients within each brain tumour grade. The different assays that might have been measured, such as protein carbonyl, were not measured. Finally, the impacts of other factors, which may affect the measured parameters, were not assessed.

## Conclusion

Our study data revealed that there was a significant imbalance in the oxidant and antioxidant statuses of brain tumour patients when compared to healthy controls. The serum concentrations of oxidants increased and antioxidants decreased in advanced tumour grades and the serum oxidants start to decrease and antioxidants increase after brain tumour patients undergo surgical therapy. The findings of this study could suggest that redox imbalance may have a role in the pathophysiology of brain tumours but further wide and experimental studies are needed to explain the relationship and possible mechanisms underlying the possible mechanisms of the decrease in antioxidants and increased oxidants levels among brain tumour patients.

## Abbreviations

BMI, body mass index; CI, confidence interval; GSH, Reduced glutathione; GSSG, Oxidised glutathione; OR, Odds ratio; ROS, Reactive oxygen species; MRI, Magnetic resonance imaging; SOD, Superoxide dismutase; TAC, Total antioxidant capacity; TOS, Total oxidative stress.

## Conflicts of interest

The authors declare that they have no competing interests.

## Ethics approval

The study was ethically approved by the Ethical Review Committee of the Biochemistry Department, College of Health Sciences, Addis Ababa University, with meeting number DRERC 08/18 and protocol number 06/19; and a formal collaboration letter was provided to all selected health centres and hospitals.

## Consent to participate

We received written informed consent from the study participants for participation, blood sample collection and for further publishing the study results. Confidentiality was maintained by omitting personal identifiers. The study protocol was conformed to the principles of the Helsinki Declaration.

## Consent to publication

Not applicable.

## Availability of data and materials

All data generated and analysed during this study are included in the manuscript.

## Funding

No funds were obtained for this particular study.

## Authors’ contributions

FT and TA were involved in the initiation of the idea, write-up of the proposal, data collection, data entry, data analysis and final manuscript write-up, while BK, BD, GY, ZG, AT, EA, AT, GW, TT, ML, TO, TJ, WA, SA, ES, MT, DG, GA, TM, DT, and TM were involved in designing the study, supervision of the research project, supervision, manuscript editing and write-up. All authors read and approved the final manuscript.

## Figures and Tables

**Table 1.** Descriptive analysis of the socio-demographic characteristics of brain tumour (BT) patients and control group at referral hospitals of Addis Ababa, Ethiopia, 2020.

Variables	Category	BT (*N* = 50) *N* (%)	Control group (*N* = 50) *N* (%)
Age (years) Mean ± SD	NA	35.25 ± 6.8	36.2 ± 7.2
Gender	Male Female	31 (62) 19 (38)	34 (68) 16 (32)
Residence	Urban Rural	25 (50) 25 (50)	25 (50) 25 (50)
Marital status	Single Married Widowed	18 (36) 29(58) 3 (6)	30 (60) 13 (26) 7 (14)
Educational level	Illiterate Primary school Secondary school College/University	21 (42) 13 (26) 4 (8) 12 (24)	18 (36) 10 (20) 10 (20) 12 (24)
Alcohol drinking habit	Yes No	26 (52) 24 (48)	14 (28) 36 (68)
Cigarette smoking habit	Yes No	0 (0) 50 (100)	7 (14) 43 (86)
Physical exercise habits	Never Once a week 2–3 times a week 4–6 times a week	31 (62.2) 4 (8.4%) 10 (20.2) 5 (11.6%)	27 (54 ) 10 (20) 10 (20) 3 (6)
Body mass index (kg/m^2^)	Underweight (<18.5) Normal weight (18.5–24.9) Overweight (25–29.9) Obese (≥30)	4 (8) 34 (68) 8 (16) 4 (8)	2 (4) 30 (60) 12(24) 6 (12)

**Table 2.** Histological and pathological characterisation of tumour tissue of BT patients attending referral hospitals in Addis Ababa, Ethiopia, 2020.

Clinicopathological profile of BTD (*N* = 50)	Category	Frequency
Size of the tumour (in cm)	Tumour length	3.6 cm ± 1.12cm
	Tumour width	2.30 cm ± 1.57 cm
Family history of BT	Yes	2 %
	No	98 %
Histologic type of tumour	Meningioma Astrocytoma Schwannoma Ependymoma Medulloblastoma Oligodendroglioma Ganglioneuroma	19 9 6 3 4 5 4
Tumour grading	Grade I	21
	Grade II	14
	Grade III	9
	Grade IV	6

BT = Brain tumour, BTD = brain tumour disease. Tumour length and width were measured in centimetre.

**Table 3.** Comparative mean analysis of serum of GSH, GSSG, TOS, TAC and OSI of BT patients and control group in referral hospitals, Addis Ababa, Ethiopia, 2020.

Serum parameters	Control group(*N* = 50)	BT patients (*N* = 50)	*p* value	95% CI
GSH	20.04 ± 2.6	11.09 ± 1.26	0.001	−9.77, −8.15
GSSG	0.21 ± 0.07	0.72 ± 0.03	0.001	0.48, 0.52
TOS	6.59 ± 0.81	9.66 ± 1.76	0.001	2.52, 3.61
TAC	118.68 ± 10.19	79.65 ± 17.91	0.017	−44.81, −33.25
OSI (ratio of TOS/TAC*100)	5.61 ± 0.92	13.37± 5.89	0.006	6.08, 9.44

Measuring units of GSH and GSSG are in μM/μg, TOS is in μmol H_2_O_2_ Eq/L, TAC is in mmol Trolox Eq/L and OSI is in arbitrary units.

**Table 4.** Comparative mean [one-way ANOVA (post-hoc)] analysis of different serum parameters across different grades identified in brain tumour patients who participated, Addis Ababa, Ethiopia, 2020.

Serum parameter	Tumour grade			
	Grade I (*N* = 21) Mean ± SD	Grade II (*N* = 14) Mean ± SD	Grade III (*N* = 9) Mean ± SD	Grade IV (*N* = 6) Mean ± SD
GSH	11.96 ± 1.13^a^	11.10 ± 0.97^a^	10.14 ± 0.22	9.46 ± 0.46
GSSG	0.69 ± 0.02^b^	0.71 ± 0.015^b^	0.75 ± 0.029	0.74 ± 0.035
TOS	8.43 ± 1.56^c^	9.46 ± 0.77^c^	11.31 ± 0.79	11.96 ± 0.72
TAC	93.80 ± 4.49^d^	84.89 ± 8.39^d^	57.89 ± 7.25^d^	50.55 ± 3.14^d^
OSI	9.11 ± 1.79^e^	11.26 ± 1.54^e^	19.87 ± 3.25^e^	23.78 ± 2.65^e^

a Significant mean difference in GSH

b Significant mean difference in GSSG

c Significant mean difference in TOS

d Significant mean difference in TAC

e Significant mean difference in OSI

**Table 5.** Comparative mean (one-way ANOVA (*post-hoc*)) analysis of the different serum parameters at different time periods at referral hospitals of Addis Ababa, Ethiopia, 2020.

Serum parameters	Comparison groups		
	Control group *N* = 50	BTP; Before surgery *N* = 50	BTP; 1 month after surgery *N* = 43
GSH	20.04 ± 2.6^a^	11.09 ± 1.26^a^	17.36 ± 2.55^a^
GSSG	0.21 ± 0.07^b^	0.72 ± 0.032^b^	0.47 ± 0.08^b^
TOS	6.59 ± 0.81^c^	9.66 ± 1.76^c^	7.79 ± 0.92^c^
TAC	118.68 ± 10.19	79.65 ± 17.91^d^	116.78 ± 5.03^d^
OSI	5.61 ± 0.92	13.37± 5.89^e^	6.68 ± 0.86^e^

a Significant mean difference in GSH

b Significant mean difference in GSSG

c Significant mean difference in TOS

d Significant mean difference in TAC

e Significant mean difference in OSI
